# Ideas of a Future Life

**Published:** 1890-04

**Authors:** 


### IDEAS OF A FUTURE LIFE.

The Iroquois and Hurons believe in a country for the souls of the dead, which they call the "country of ancestors." This is the west, from which direction their traditions told that they had migrated. Spirits must go there after death by a very long and painful journey, past many rivers, and at the end of a narrow bridge fight with dog-like Cerberus, and some may fall into the water and be carried away over precipices. This road is all on the earth; but several of the Indian tribes consider the Milky Way to be the path of souls, those of human beings forming the main body of the stars, and their dogs, which also have souls, running on the sides. In their next world the Indians do the same as they customarily do here, but without life's troubles.

The Israelites believed in a doubling of the person by a shadow, a pale figure, which after death descended under the earth and there led a sad and gloomy existence. The abode of these poor beings was called Sheol. There was no recompense, no punishment. The greatest comfort was to be among ancestors and resting with them. There were some very virtuous men whom God carried up that they might be with him. Apart from these elect, the dead went into torpor. Man's good fortune was to be accorded a long term of years, with children to perpetuate his family and respect for his memory after death.—*Popular Science Monthly.*

